# Respiratory and behavioral dysfunction following loss of the GABA_A_ receptor α4 subunit

**DOI:** 10.1002/brb3.122

**Published:** 2013-02-05

**Authors:** C Jean Loria, Ashley M Stevens, Ellen Crummy, Gemma Casadesus, Frank J Jacono, Thomas E Dick, Ruth E Siegel

**Affiliations:** 1 Department of Pharmacology, Case Western Reserve University10900 Euclid Avenue, Cleveland, Ohio, 44106; 2 Department of Neurosciences, Case Western Reserve University10900 Euclid Avenue, Cleveland, Ohio, 44106; 3 Department of Medicine, Case Western Reserve University10900 Euclid Avenue, Cleveland, Ohio, 44106; 4 Louis Stokes Cleveland VA Medical Center10701 East Blvd., Cleveland, Ohio, 44106

**Keywords:** Control of breathing, GABA_A_ receptor subunits, pons, ventilatory response

## Abstract

γ-Aminobutyric acid type A (GABA_A_) receptor plasticity participates in mediating adaptation to environmental change. Previous studies in rats demonstrated that extrasynaptic GABA_A_ receptor subunits and receptors in the pons, a brainstem region involved in respiratory control, are upregulated by exposure to sustained hypobaric hypoxia. In these animals, expression of the mRNA encoding the extrasynaptic α4 subunit rose after 3 days in sustained hypoxia, while those encoding the α6 and δ subunits increased dramatically by 2 weeks. However, the participation of extrasynaptic subunits in maintaining respiration in normoxic conditions remains unknown. To examine the importance of α4 in a normal environment, respiratory function, motor and anxiety-like behaviors, and expression of other GABA_A_ receptor subunit mRNAs were compared in wild-type (WT) and α4 subunit-deficient mice. Loss of the α4 subunit did not impact frequency, but did lead to reduced ventilatory pattern variability. In addition, mice lacking the subunit exhibited increased anxiety-like behavior. Finally, α4 subunit loss resulted in reduced expression of other extrasynaptic (α6 and δ) subunit mRNAs in the pons without altering those encoding the most prominent synaptic subunits. These findings on subunit-deficient mice maintained in normoxia, in conjunction with earlier findings on animals maintained in chronic hypoxia, suggest that the expression and regulation of extrasynaptic GABA_A_ receptor subunits in the pons is interdependent and that their levels influence respiratory control as well as adaptation to stress.

## Introduction

The ability to rapidly adapt to environmental fluctuations is essential for maintaining respiratory homeostasis. While much has been learned about signaling pathways involved in regulating adaptation to different hypoxic conditions (Papandreou et al. 2005), including sustained hypobaric hypoxia (Storz et al. 2010), the circuitry and mechanisms involved in maintaining respiratory control in normoxia are less well characterized.

GABAergic signaling is one pathway that has been implicated in respiratory control (Hedner et al. 1981). Many of the actions of γ-aminobutyric acid (GABA), the major inhibitory neurotransmitter in the brain, are mediated by pentameric GABA_A_ receptors assembled from a pool of 19 subunits. The function of these receptors depends on subunit composition (reviewed in Sieghart 2006; Olsen and Sieghart 2009; Uusi-Oukari and Korpi 2010). The most abundantly expressed subunits, α1, β2, and γ2, contribute to synaptic receptors that mediate the rapid, phasic effects of GABA throughout the brain. Other subunits, including α4, α6, and δ, contribute predominantly to extrasynaptic or perisynaptic receptors that are distributed less widely. These receptors mediate the tonic effects of low concentrations of ambient GABA (Farrant and Nusser 2005; Walker and Semyanov 2008).

Previous studies demonstrated that sustained exposure to hypobaric hypoxia was accompanied by altered GABA_A_ receptor expression in the pons (Hsieh et al. 2004, 2008), a brainstem region that participates in the control of respiratory behavior (Alheid et al. 2004; Chamberlin 2004; McCrimmon et al. 2004; Smith et al. 2009). The level of the extrasynaptic α4 subunit mRNA rose most rapidly, becoming maximal after 3 days of exposure. Increased or de novo expression of two other extrasynaptic subunits, δ and α6, was detected after 2 weeks of exposure (Hsieh et al. 2004, 2008). These increases in subunit mRNA expression were accompanied by increases in extrasynaptic receptor number (Hsieh et al. 2008). The plasticity of extrasynaptic GABA_A_ receptor subunits and receptor levels in response to a respiratory insult raises the possibility that extrasynaptic receptors are essential for maintaining respiratory function in normoxic conditions.

To investigate the role of α4 subunit expression in a normoxic environment, the respiratory patterns, motor and anxiety-like behaviors, and subunit expression were compared in mice lacking this subunit (Gabra4^−/−^; knockout [KO]) and wild-type (WT) littermates (Gabra4^+/+^; WT). Studies of respiratory function demonstrated that deletion of α4 had no impact on respiratory rate, but reduced the variability of the ventilatory pattern. This physiologic change was accompanied by increased anxiety-like behavior. In addition, compensatory age-dependent changes in the expression of some receptor subunit mRNAs in the pons were observed. While levels of the mRNAs encoding the most abundant synaptic subunits were unaltered in the KO mice, levels of mRNAs encoding extrasynaptic subunits were reduced. In conjunction with our previous findings concerning the effects of sustained hypoxia, these results suggest that the balance of synaptic and extrasynaptic receptor activity, and presumably cross-talk between GABAergic signaling and other neurotransmitter systems, is necessary to maintain respiratory behavior.

## Methods

### Animals

Heterozygous GABA_A_ receptor α4 subunit-deficient mice generated on a 129X1/S1 × C57BL/6J genetic background (gift of G. Homanics; Chandra et al. 2006) were cross-foster rederived using C57Bl/6J animals. GABA_A_ receptor α4 subunit-deficient (Gabra4^−/−^; KO), heterozygous (Gabra4^+/−^), and WT littermate (Gabra4^+/+^) mice were then produced from heterozygous rederived breeding pairs (Gabra4^+/−^) of the F8+ generations. All experiments were performed using 1- to 3-month-old WT or KO mice that were housed in the Case Western Reserve University (CWRU) Animal Facility on a 12-h light/12-h dark cycle. Male and female mice were used because gender differences were not observed in this or previous studies (Chandra et al. 2008). All procedures and protocols were approved by the Institutional Animal Care and Use Committee of CWRU.

### Measurement of respiratory parameters

Ventilatory measurements were made on unrestrained, unanesthetized 60- to 75-day-old WT and KO mice using a whole-body plethysmograph (Buxco Plethysmograph, NC). Experiments were initiated between noon and 2:00 pm to limit the influence of circadian rhythm; the laboratory environment was climate controlled and variations in room temperature were minimal. The animals were allowed at least 1 h to acclimate to the plethysmograph chamber. Pressure changes in the chamber associated with air flowing in and out of the animal were sensed by a differential pressure transducer (TRD5700; Buxco Electronics) connected to the recording and reference chambers; the two chambers were connected through a high-resistance port to compensate for slow pressure changes. The plethysmograph was calibrated with 0.1 mL air injected and removed at ∼1.5 Hz, to present data as an estimate of tidal volume (*V*_t_). No correction was made for the animal's body temperature during the recording period. The signal from the transducer was amplified with a preamplifier (Max II; Buxco Electronics), analog-to-digital converted and acquired (sampling rate = 200 Hz) with a computer interface (Power1401; CED, Cambridge, U.K.) and stored using data acquisition software (Spike 2; CED) for off-line analysis of ventilatory pattern dynamics.

### Respiratory data analysis

Breathing pattern measurements were the mean for breaths in 30 sec. Variables measured included peak amplitude (PK; millivolts indicating change in inspiratory *V*_t_), respiratory frequency (FR; breaths/min), inspiratory time (TI), and expiratory time (TE). Breath-to-breath variabilities of TI, TE, and FR were assessed by calculating the coefficient of variation ([standard deviation/mean] × 100). In addition, Poincaré analysis was used to quantify variability in TI and TE as described previously (Jacono et al. 2010; Fishman et al. 2012). Poincaré return maps were constructed by plotting each respiratory cycle length (*n* + 1) against the previous one (*n*). Visual inspection revealed symmetric clusters of points, and the “ellipse fitting” method was used to quantify short-term variability (SD1) and long-term variability (SD2) (Tulppo et al. 1996; Brennan et al. 2002; Fishman et al. 2012) using Software-R, MatLab (Gonsenhauser et al. 2004). A two-way repeated-measures analysis of variance (ANOVA) was used to test for significance of differences between WT and KO mice. Specific differences were identified by Student-Newman–Keuls test. Results are expressed as means ± SD.

### Behavioral assays

A number of behavioral tests were performed using free-fed, gender- and age-matched (8- to 12-week) mice.

Motor behavior was assayed using the treadmill assay. WT and KO mice were familiarized with treadmill running prior to measuring exercise endurance using a rodent Exer3/6 treadmill (Columbus Instruments, Columbus, OH). On day 1 of habituation, mice were allowed to explore the treadmill freely for 10 min. On day 2, animals were habituated to walking at 10 m/min for 10 min, and on day 3, the speed was increased to 12 m/min. In the testing sessions, the mice were required to walk at a relatively easy pace of 10 m/min for 10 min before increasing the pace to 20 m/min in 2-min intervals, a standard exercise running test. To encourage the mice to run, the treadmill was equipped with an electrical shock grid at the rear of the treadmill that delivered a shock (0.15 mA) that did not harm or injure the animals. When the mice reached exhaustion, as defined by their inability to run for 10 sec, testing was discontinued.

Home cage activity was measured for 21 h to determine the activity patterns of the WT and KO mice in their normal habitat without experimenter interference. Locomotor activity was measured by tracking the animals using a high-definition CCD camera (Panasonic, Osaka, Japan) and tracking system software (Ethovision XT, Noldus, Wageningen, NL). Animal activity was tracked during the light and dark phases and during transitions between the two phases.

Anxiety-related behavior in WT and KO mice was compared using an automated elevated plus-maze (Med Associates, VT). The maze consists of a platform and four arms, two of which are enclosed. The animals can see their high elevation in the open, but not the closed arms. The animals were placed in the center facing an enclosed arm and allowed to explore freely. Animal activity was tracked for 5 min; the number of explorations (defined as entrance of only the front paws), entries (defined as entrance with all four paws), and time spent in the open arms, the closed arms, or in the central platform were recorded.

### RNA preparation and quantitative real-time PCR

Brains were rapidly removed from euthanized mice and frozen at −80°C until use. The pons, medulla, cerebellum, cortex, thalamus, hippocampus, and olfactory bulbs were dissected from the brains of at least three mice at each age, and RNA was extracted using Trizol (Life Technologies, Carlsbad, CA). Samples were also prepared from the liver, a tissue lacking GABA_A_ receptors, to confirm probe specificity.

Expression of mRNAs was assessed by the quantitative real-time polymerase chain reaction (q-PCR). Complementary DNAs (cDNAs) were generated from the RNAs using the High Capacity RNA-to-DNA kit (Life Technologies). Real-time q-PCR was carried out using TaqMan chemistry and Assays-on-Demand probes (Applied Biosystems) for the GABA_A_ receptor α1 (Mm00439046_m1), α2 (Mm00433435_m1), α4 (Mm00802631_m1), α5 (Mm00621092_m1), α6 (Mm01227754_m1), β2 (Mm00633467_m1), γ2 (Mm00433489_m1), δ (Mm00433476_m1), and ε (Mm00489932_m1) subunits. Additional assays were performed for glutamic acid decarboxylase 65 (GAD65; Mm00484623_m1), and GAD67 (Mm00725661_s1), enzymes important in GABA synthesis, and gephyrin (Mm00556895_m1), a molecule participating in receptor clustering at the synapse. 18S rRNA (4352930E) was used as an internal standard.

### RNA data analysis

Assays were all performed in triplicate using Applied Biosystems Step-One Plus Real-Time PCR system. The *C*_T_ (cycle number at threshold) was used to calculate relative mRNA amounts (Livak and Schmittgen 2001). The *C*_T_ of each target gene was normalized by subtracting the *C*_T_ value of 18S RNA, the housekeeping gene, which gave the value Δ*C*_T_. Values are expressed as 2^−ΔCT^ and are normalized to reference samples as indicated. Data from WT and KO animals are reported here. We also analyzed Gabra4^+/−^ mice and found gene expression levels similar to WT (not shown).

## Results

### Characteristics of GABA_A_ receptor α4 subunit-deficient mice

Previous studies demonstrated that Gabra4^−/−^ mice were viable, bred normally, and were similar in weight to WT littermates (Chandra et al. 2006). These characteristics were maintained in the rederived KO animals used in our studies. No significant differences in weight between WT and KO mice were found, and only background levels of the α4 subunit were detected immunohistochemically in the KO brain. Finally, brain morphology was similar in WT and KO mice ranging from 30 to 90 days (P30–90) in age (data not shown).

### Loss of the GABA_A_ receptor α4 subunit results in decreased ventilatory pattern variability

**Figure 1:**
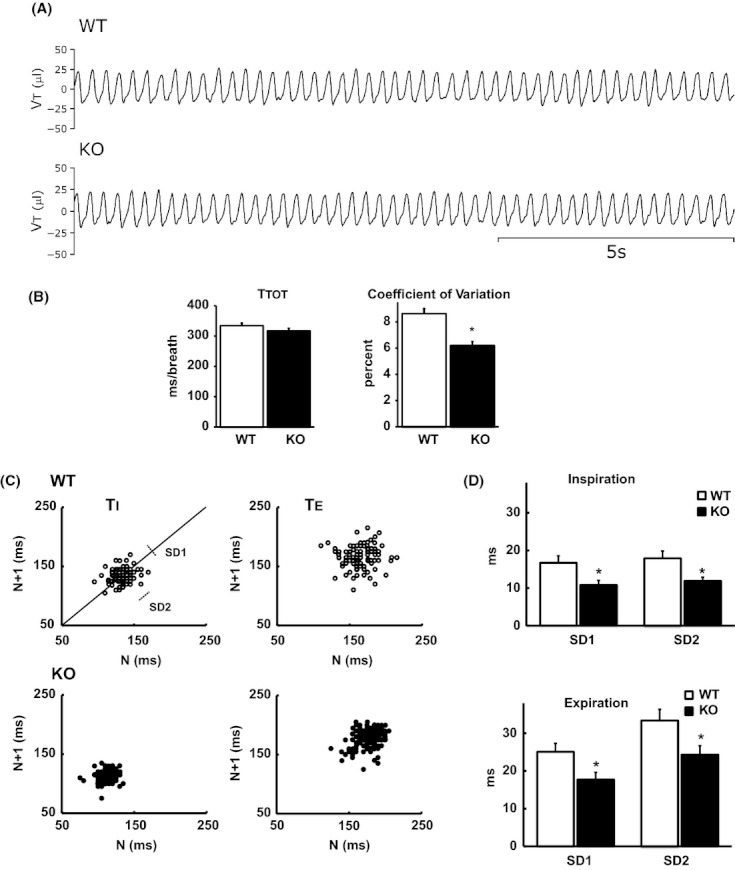
The variability of respiration is reduced in α4 subunit-deficient mice. (A) Representative respiratory traces show that the total time of the respiratory cycle (TTOT) is similar (∼300 msec/breath) for a wild-type (WT) (top) and an α4 subunit-deficient mouse (bottom). Traces were obtained using flow-through whole-animal plethysmography. Inspiration is upward on the trace. (B) TTOT (left) was not significantly different in WT (*n* = 16) and knockout (KO; *n* = 13) animals. The median line is close to the value for the third quartile for the KO animals, indicating that the data were skewed toward the higher value for TTOT. In contrast, the coefficient of variation (right) for KO mice was significantly less than that for WT animals. *P < 0.01. (C) Poincaré analysis shows the distribution of inspiration (TI) and expiration (TE) times for WT and α4 KO animals. The values for TI and TE for the current breath (*N*) were plotted against those values in the next breath (*n* + 1). Successive points with equal values fall along the 45º diagonal, the line of identity. (D) Variability of the data in the Poincaré plots was estimated by calculating the standard deviations normal to the diagonal (SD1) and along the diagonal (SD2). The former measures short-term variability and the latter measures long-term variability; *P < 0.01. SD1 and SD2 were analyzed separately for each animal. The means ± SD are shown.

To test the possibility that global loss of the GABA_A_ receptor α4 subunit affects respiration, ventilatory wave forms were recorded from spontaneously breathing, unrestrained WT (*n* = 13) and KO (*n* = 16) mice using flow-through plethysmography. Representative traces (Fig. 1A) show that the ventilatory patterns of WT and KO mice were similar. Further analysis revealed that total time of the respiratory cycle (TTOT) for all mice was comparable (∼300 msec/breath; Fig. 1B).

Although the respiratory rates were similar, quantitative analysis of the ventilatory pattern demonstrated that it was less variable in KO mice. Analysis of TTOT showed that the coefficient of variation was significantly lower in KO than in WT animals (Fig. 1B). Further examination of breath-to-breath variation using Poincaré analysis (Fig. 1) demonstrated that the inspiration (TI) and expiration (TE) times were more tightly clustered in KO than in WT animals (Fig. 1C and D). To quantify the respiratory variability, we calculated SD1 and SD2 for the inspiratory (TI) and expiratory (TE) times. SD1, which is computed from variation of the normal to the diagonal, is a measure of breath-to-breath variability; SD2, which is computed from variation along the diagonal, is a measure of long-term variation. This analysis shows that both TI and TE had significantly less long- and short-term variability in KO mice.

### Deletion of the GABA_A_ receptor α4 subunit results in altered anxiety-like behavior

To test the possibility that alterations in the respiratory pattern were a consequence of changes in motor function, the physical endurance of WT and KO mice was compared using a motor-driven treadmill. In the first set of assays, the KO mice (*n* = 4) failed to perform. In contrast to the WT mice (*n* = 3), these mice refused to run during the training sessions and could not be induced to remain on the treadmill. To determine whether this failure reflected a true motor deficit, a second test was performed using different KO and WT mice. In this assay, the KO mice (*n* = 4) ran, but at speeds approximately 25% slower than those of WT mice (*n* = 3); quantification of their performance demonstrated that their endurance was reduced by 12%. Despite this decrease, the KO mice did not exhibit obvious motor deficits.

**Figure 2:**
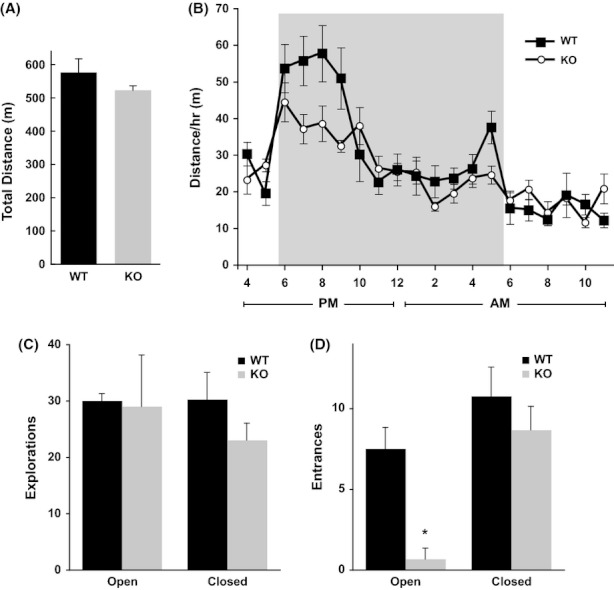
Loss of γ-aminobutyric acid (GABA_A_) receptor α4 subunit alters emotional behavior. (A) Open-cage activity test demonstrates that knockout (KO) and wild-type (WT) mice traveled similar distances during the 21-h assay period. (B) The KO mice were less mobile than WT mice during periods of transition from light to dark or the reverse. Shaded box represents the dark phase. (C) Elevated-plus maze test demonstrates that KO and WT mice exhibited similar exploratory behavior at the open and closed arms of the maze. (D) In contrast, the KO mice were significantly less likely to enter the open arms than WT mice. Vertical bars represent means ± SEM (*n* = 4). *P < 0.01.

To further assess motor function, activity of WT and KO mice in the home cage was assessed. These studies demonstrated that the movement of WT and KO mice over a period of 21 h was similar (Fig. 2A). While the mice traveled similar distances in both light and dark environments (Fig. 2B), the pattern differed slightly. The WT mice were more active during the transitions between the light and dark environments, suggesting differences in circadian rhythm or in the response to environmental stimuli.

Additional evidence that motor activity was not altered was obtained by comparing the behavior of WT and KO mice in the elevated plus maze. In these assays, the animals were placed in the center of an elevated four-arm maze, which had two open and two closed (protected) arms. In this assay, the WT and KO mice explored both the open and closed arms of the maze a similar number of times (Fig. 2C). However, the KO and WT mice showed a striking difference in their entries into the arms of the maze (Fig. 2D). WT mice entered the open and closed arms a similar number of times, but the KO mice showed a strong preference for the closed arms. Avoidance of the open arms and decreased entries are symptomatic of increased anxiety. Together, these behavioral tests suggest that the α4 subunit-deficient mice exhibit increased anxiety-like behavior in the absence of major changes in motor function.

### Levels of other extrasynaptic GABA_A_ receptor subunit mRNAs in the pons are altered in α4 KO mice

**Figure 3:**
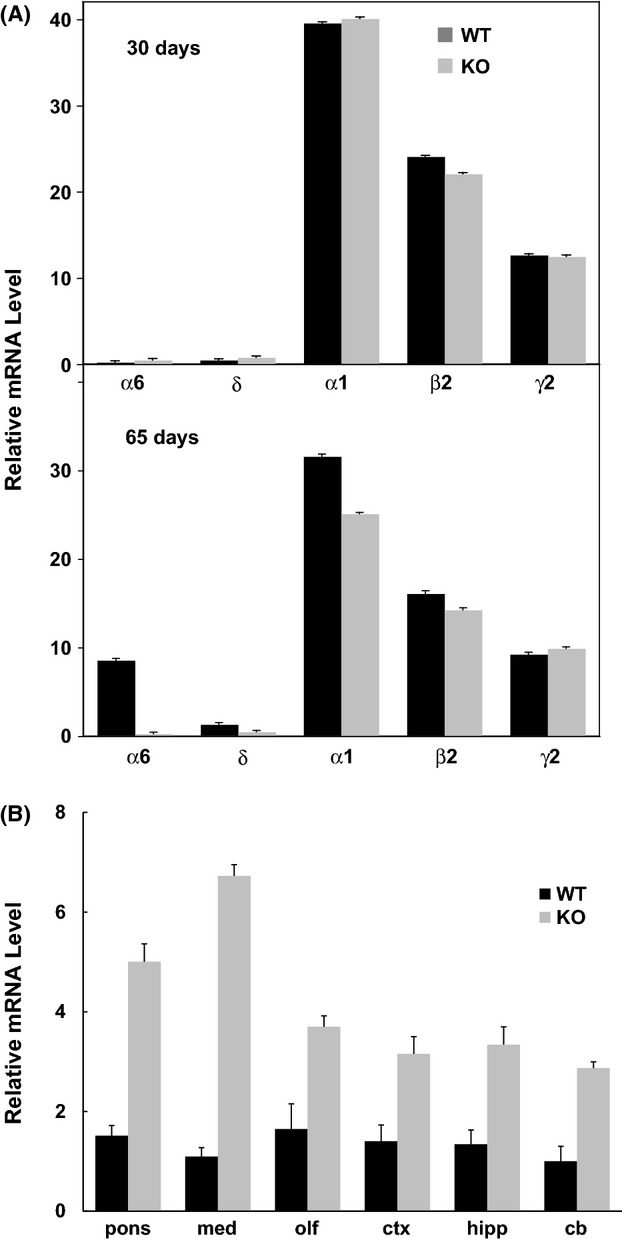
Loss of γ-aminobutyric acid (GABA_A_) receptor α4 subunit affects the expression of other GABA_A_ receptor subunits. (A) Quantitative real-time polymerase chain reaction (q-PCR) analysis of abundant synaptic and extrasynaptic subunit mRNAs in the pons of wild-type (WT) and knockout (KO) mice at postnatal days (P) 30 and 65. (B) q-PCR analysis of the expression of α2, a less prominent synaptic mRNA, in the indicated brain regions of WT and KO mice at P60. mRNAs were analyzed from pons, medulla (med), olfactory bulb (olf), cerebral cortex (ctx), hippocampus (hipp), and cerebellum (cb). Results are expressed as the ratio of mRNA to 18S rRNA in each sample. Error bars represent the standard error of the mean (SEM, *n* = 3).

Previous studies found that subunit loss can lead to compensatory changes in the expression of other subunits in a region-specific manner (Ogris et al. 2006; Liang et al. 2008). To further examine the selectivity of the α4 subunit in regulating respiratory function, subunit expression was examined in WT and KO mice in the pons, a brainstem region involved in respiratory control. In WT mice, extrasynaptic GABA_A_ receptor subunit expression was age dependent. The α6 subunit mRNA was undetectable at P30, but was found at relatively low levels at P65 (Fig. 3A). Similarly, the δ subunit mRNA was absent at P30, and low levels were found by P65. In contrast to WT mice, extrasynaptic subunit expression in α4 subunit KO mice was not age dependent. Both the α6 and δ subunit mRNAs were barely detectable at all ages examined (Fig. 3A). Despite this lack of the of the most prominent extrasynaptic subunits, compensatory increases in the expression of mRNAs encoding α5 and ε, two other less abundant extrasynaptic subunits, were not observed (not shown).

In contrast to the impact of α4 subunit loss on other extrasynaptic subunits, synaptic subunit expression was only modestly affected. The levels of the mRNAs encoding the most abundant synaptic subunits, α1, β2, and γ2, were similar in WT and KO mice at postnatal days P30 and P65 (Fig. 3A) as well as P90 (not shown). In contrast, the level of a sparse synaptic subunit, α2, was differentially affected in the WT and KO mice (Fig. 3B). While all mice expressed the subunit in the pons, the loss of α4 led to a three- to fivefold increase in its expression. In addition, although α2 subunit expression increased with age in all mice, the change was greater in KO animals. A similar age-dependent increase of α2 subunit expression was found in other brain regions (not shown). Thus, loss of α4 leads to genotype-, age-, and tissue-dependent changes in the expression of both synaptic and extrasynaptic GABA_A_ receptors subunits. It is likely that these differences in subunit expression in WT and KO mice affect GABA_A_ receptor composition and alter the balance of synaptic and extrasynaptic receptors (Hsieh et al. 2004), thus modifying GABAergic signaling and cross-talk with other neurotransmitter systems and the regulation of respiratory control.

**Figure 4:**
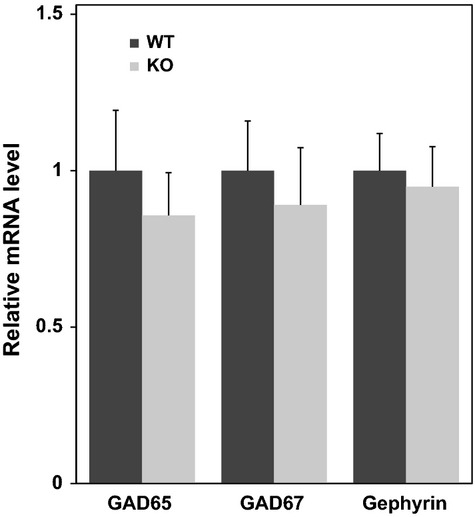
Glutamic acid decarboxylase (GAD) 65, GAD67, and gephyrin mRNA expression in the pons of α4 subunit-deficient mice is not altered by subunit loss. mRNA from the pons of wild-type (WT) and knockout (KO) mice was assessed by quantitative real-time polymerase chain reaction (q-PCR). Results are expressed as the ratio of mRNA to 18S rRNA in each sample and are normalized to the corresponding value from WT animals. Error bars represent the standard error of the mean (SEM, *n* = 3). Similar levels of both GAD mRNA isoforms and gephyrin were found in mutant and WT mice.

To examine whether other factors influence the differences in subunit expression in WT and KO mice, several studies were performed. Recent findings suggest that the neurotransmitter, GABA, can participate in regulating the plasticity of inhibitory synapses in mature animals as well as in mediating signaling (Huang 2009). To assess the role of the neurotransmitter in subunit expression in the pons, the expression of GAD, the enzyme that converts glutamic acid to GABA, was compared in WT and KO mice. There are two GAD isoforms (Kaufman et al. 1991); one resides primarily in the synapse (GAD67) and the other is found throughout the cytoplasm (GAD65). Our studies demonstrated that the levels of both GAD mRNAs were virtually identical in the pons (Fig. 4) and cerebellum (not shown) of the WT and KO mice at any age. These results suggest that the observed differences in GABA_A_ receptor subunit expression are unlikely to be a consequence of changes in neurotransmitter level.

In addition, receptor assembly and plasticity can be shaped by subunit interactions with a variety of scaffolding proteins. One protein that has been shown to bind directly or indirectly with inhibitory receptors to maintain their stability in the plasma membrane is gephyrin (Kneussel and Loebrich 2007; Fritschy et al. 2008; Tretter and Moss 2008). This protein is also unlikely to play a role as the levels of mRNAs encoding gephyrin were similar in the pons (Fig. 4) and cerebellum (not shown) in WT and KO mice.

## Discussion

Global loss of the GABA_A_ receptor α4 subunit led to alterations in the respiratory pattern in mice maintained in a normoxic environment. While respiratory rates were similar in the KO and WT mice, breath-to-breath variability was significantly decreased in the subunit-deficient animals. Moreover, Poincaré analysis showed that there was a reduction in both long- and short-term variability of inspiration and expiration. This respiratory change occurred in mice lacking the α4 subunit as well as decreased expression of other extrasynaptic subunits. In conjunction with previous findings in rats maintained in sustained hypoxia (Hsieh et al. 2004, 2008), these findings raise the possibility that multiple extrasynaptic subunits and the balance of synaptic and extrasynaptic receptors in the brainstem may be involved in maintaining the respiratory rhythm and the plasticity of ventilatory behavior. These findings also suggest that extrasynaptic GABA_A_ receptor subunit expression is interdependent. These issues will be further addressed using mice lacking other extrasynaptic subunits. Such KO mice will provide important models for determining whether specific extrasynaptic subunits are sufficient for mediating the response to acute hypoxic insults or for adaptation to sustained hypoxia.

Previous studies have shown that the brainstem is involved in the control of respiratory function (Guthmann et al. 1998; Frazao et al. 2007; Waldvogel et al. 2010). Our studies demonstrate that loss of α4 from the pons is accompanied by induction of the mRNAs encoding α2, a synaptic subunit, and by decreases in expression of two extrasynaptic subunits, α6 and δ. Finding α6 in the pons of WT mice was surprising because this subunit previously was found almost exclusively in postnatal cerebellar granule neurons (Kato 1990; Luddens et al. 1990). A recent report, however, similarly detected α6 in pons of humans (Waldvogel et al. 2010), suggesting that our findings in mice reflect the enhanced sensitivity of the q-PCR approach. In contrast to the observed changes in subunit expression in the pons, the loss of α4 failed to influence the expression of most subunits in the medulla, another brainstem region involved in respiratory control. This difference supports the importance of subunit expression in the pons on respiratory function.

Synaptic and extrasynaptic GABA_A_ receptors differ in function as well as subunit composition (Nusser et al. 1998; Brickley et al. 1999; Mody 2001; Tretter et al. 2008; Walker and Semyanov 2008). Whereas synaptic receptors are transiently and rapidly activated by neurotransmitter release in the nerve terminal, extrasynaptic receptors are tonically activated by ambient GABA, which leads to the prolongation of inhibitory postsynaptic activity. Our findings suggest that the number and/or subunit composition of synaptic and extrasynaptic receptors in the pons is modified (synaptic) or downregulated (extrasynaptic) in α4-deficient mice. Such changes might perturb phasic and tonic GABAergic activity, as has been found in other brain regions of α4 subunit KO mice (Chandra et al. 2006; Liang et al. 2008). A change in inhibitory receptor signaling might alter the balance between inhibitory and excitatory activity in the brainstem. Changes in receptor expression and interaction in the pons could impact signaling to the medulla to regulate respiratory function.

The mechanisms by which changes in GABA_A_ receptor subunit expression and receptor signaling lead to differences in the respiratory patterns of the KO and WT mice, however, remain unknown. Previous studies have shown that the lateral pons contains neurons that have respiratory-modulated activity that alter the respiratory pattern when stimulated. Breath-to-breath variability can be influenced by sensory input (Bruce 1997). For example, breath-to-breath variability is decreased and respiratory drive is increased in response to increased carbon dioxide (Eldridge et al. 1989). In contrast, breath-to-breath variability is increased by vagal input in the absence of a change in respiratory drive (Sammon and Bruce 1991). Our findings suggest that loss of the GABA_A_ receptor α4 subunit in the pons affects neuronal activity involved in the modulation of the respiratory pattern. The observed decrease in respiratory variability in KO animals could result from altered signaling from the pons to medullary areas that are thought to generate the respiratory pattern.

Other studies have shown that GABA can affect the respiratory pattern (reviewed in Zuperku and McCrimmon 2002). These studies have demonstrated that a tonic GABAergic input decreases the activity of respiratory-modulated neurons in the medulla. This inhibition by GABA decreases the discharge frequency of these neurons by 50–65% and can act both phasically and tonically. Our data from the α4 subunit KO mice suggest that a decrease in tonic inhibition due to a reduction in extrasynaptic GABA_a_ receptor number augments respiratory-modulated neuronal activity to stabilize the breath-to-breath pattern.

In addition to respiratory changes, our studies also showed that loss of the α4 subunit in KO mice results in enhanced anxiety-like behavior. Most notably, KO mice explored all arms of an elevated plus maze, but almost exclusively entered the closed arms. This behavior suggests that the KO mice prefer dark, enclosed spaces (approach) and fear elevated/open spaces (avoidance). In contrast to our findings, this behavioral difference was not detected in another study using α4 subunit KO mice (Chandra et al. 2008). This disparity presumably reflects differences in the background strains of the subunit-deficient mice, animal age, or differences in the testing protocols.

The mechanisms underlying the behavioral changes in our α4 KO mice exposed to stress are difficult to identify in an intact animal. These changes could be due directly to the loss of α4 and the resulting alterations in the balance of tonic and phasic signaling in the brain. The changes may also be due to differences in the assembly of functional receptors in different brain regions or to changes in receptor–receptor interactions. Alternatively, they may be an indirect consequence of increased α2 subunit expression and changes in synaptic receptor levels in the limbic system, a brain region associated with anxiety-like behaviors (Dubois et al. 2006). Although some studies found that α2 subunit expression is reduced in anxiety-related behaviors (Nehrenberg et al. 2009), it is possible that alterations in the interaction of GABAergic and glutamatergic signaling might disrupt the balance of inhibitory and excitatory activity (Mozhui et al. 2010).

A number of studies have demonstrated that anxiety-like behavior is often associated with increased FR (Masaoka and Homma 2000, 2004; Nardi et al. 2009). The concomitant changes in these behaviors have led some to hypothesize that respiratory function and anxiety are linked by a common neural circuitry (Abelson et al. 2010). Although we did not detect changes in FR, respiratory variability decreased in the KO mice. Further studies will be necessary to examine a possible link between these behaviors.

The observed changes in subunit expression in the pons are likely consequences of alterations in subunit synthesis, trafficking from intracellular compartments, or anchoring in the membrane. A number of scaffolding proteins that anchor GABA_A_ receptor subunits have been identified. Gephyrin, a scaffolding protein that interacts primarily with subunits of synaptic receptors (Kneussel and Loebrich 2007; Renner et al. 2008; Tretter and Moss 2008), is one participant in this process in some brain regions (Waldvogel et al. 2010). The fact that gephyrin mRNA levels are comparable in the pons of WT and α4 subunit-deficient mice suggests that changes in receptor expression in this brain region occur independently of this protein. Additional studies are necessary to confirm that gephyrin protein levels are also unchanged and to determine whether the expression of other identified synaptic or extrasynaptic receptor interacting proteins is altered.

In conclusion, our studies demonstrate that loss of the GABA_A_ receptor α4 subunit modifies respiratory and anxiety-like behaviors. Accompanying these behavioral changes, the expression of GABA_A_ receptor subunits is altered; these changes presumably affect the balance between phasic and tonic GABAergic inhibition as well as that between inhibitory and excitatory signaling. Such adjustments in network activity may underlie the observed alterations in the respiratory pattern and the increased anxiety-like behavior in α4 subunit-deficient mice.
